# Supplementary figures and images for: Expression of type I collagen in response to Isoniazid exposure is indirect and is facilitated by collateral induction of cytochrome P450 2E1: An in-vitro study
Source: PLoS One. 2020 Jul 31;15(7):e0236992. doi: 10.1371/journal.pone.0236992 (PMC7394448; doi:10.1371/journal.pone.0236992)

**Figure 1B**

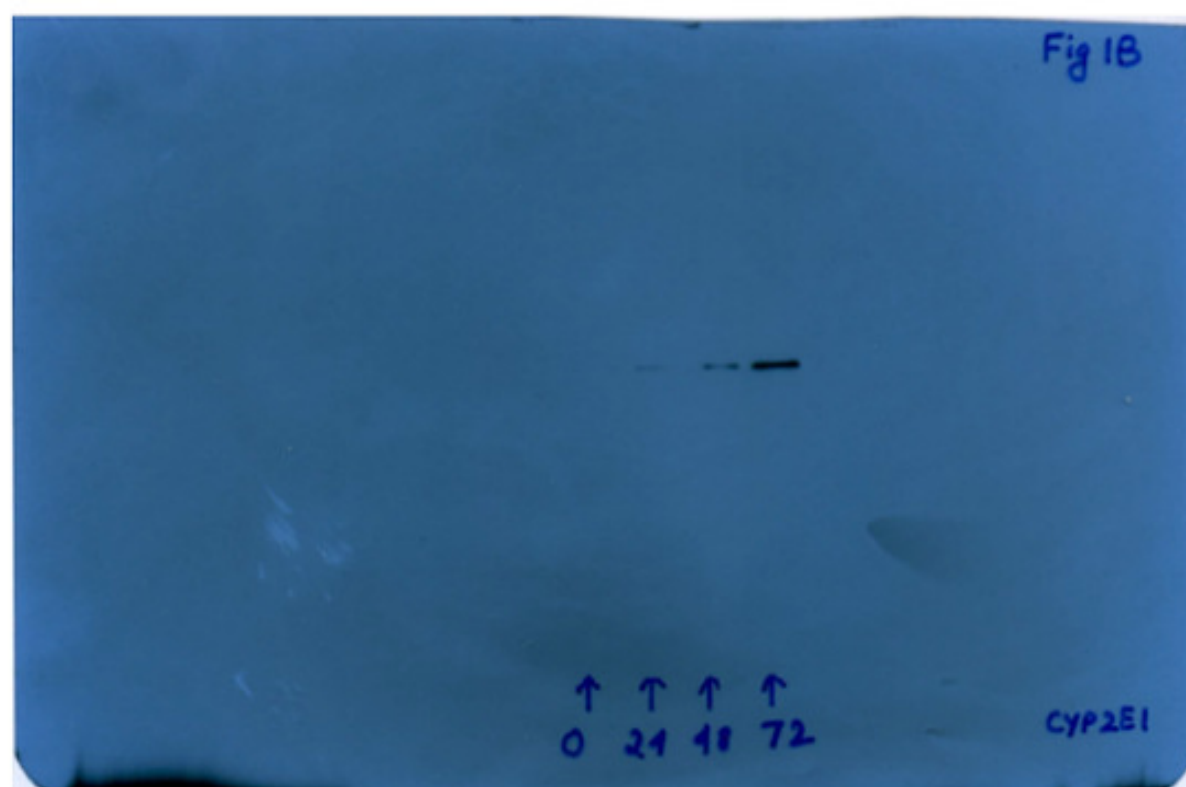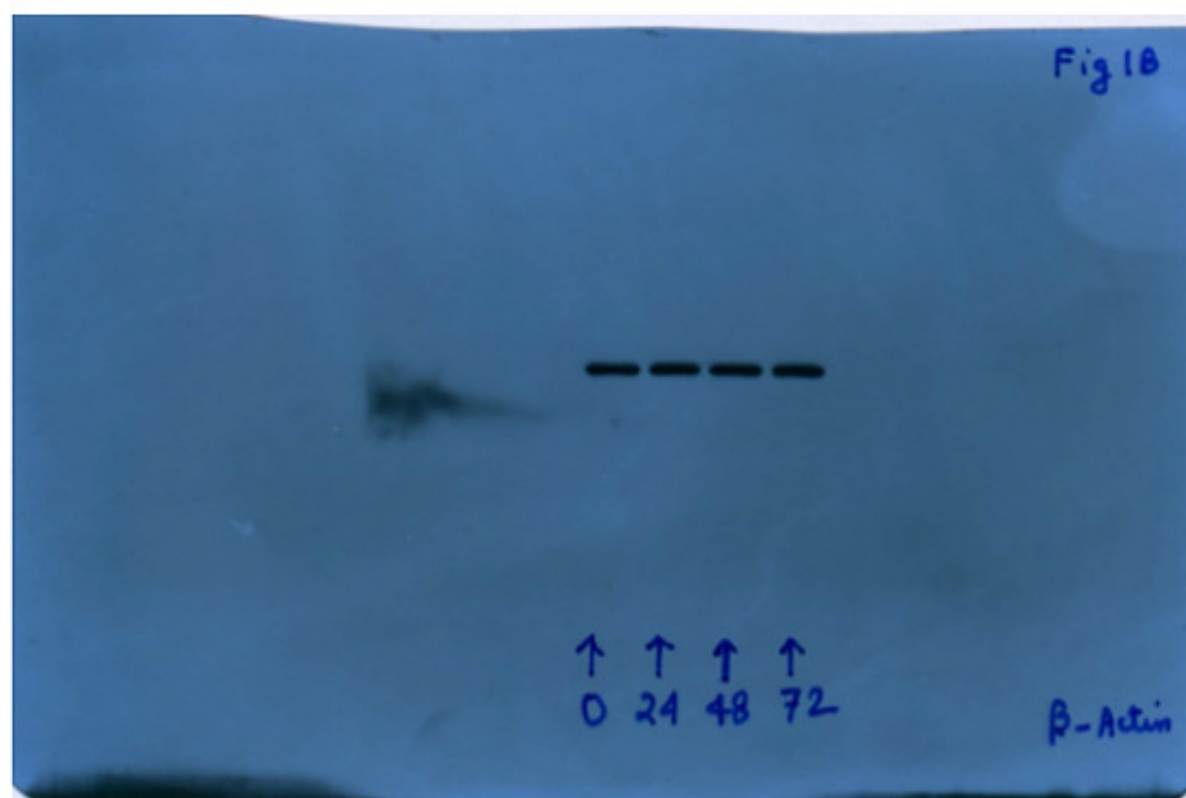

**Figure 1D**

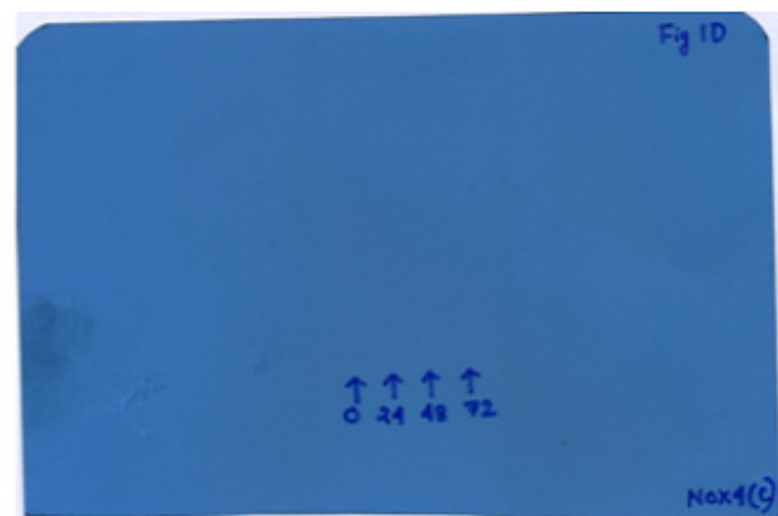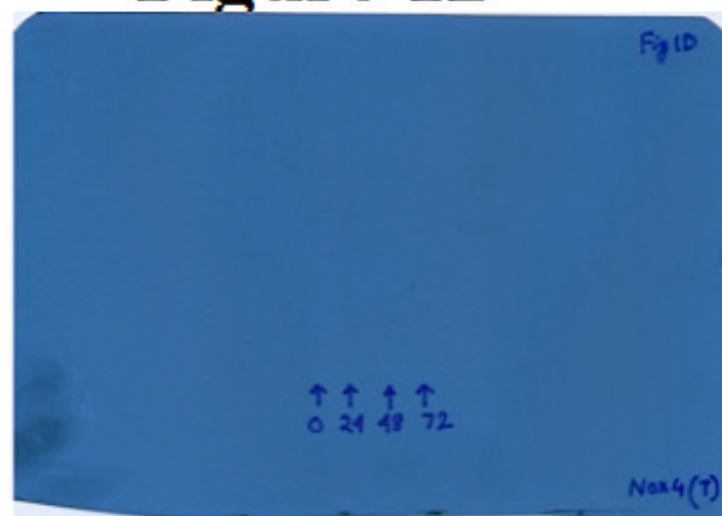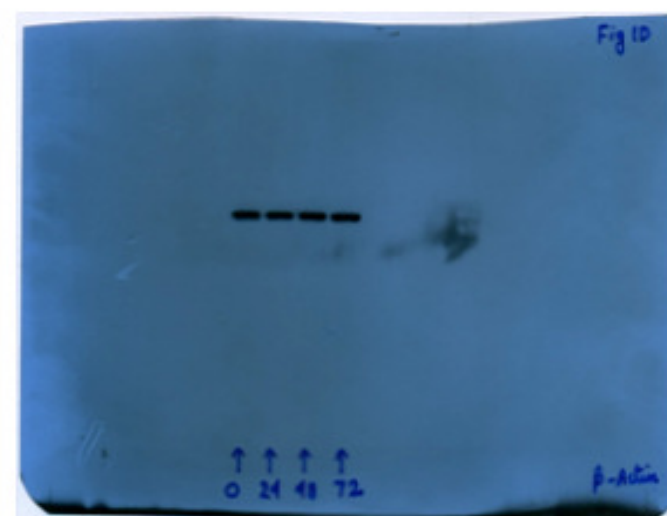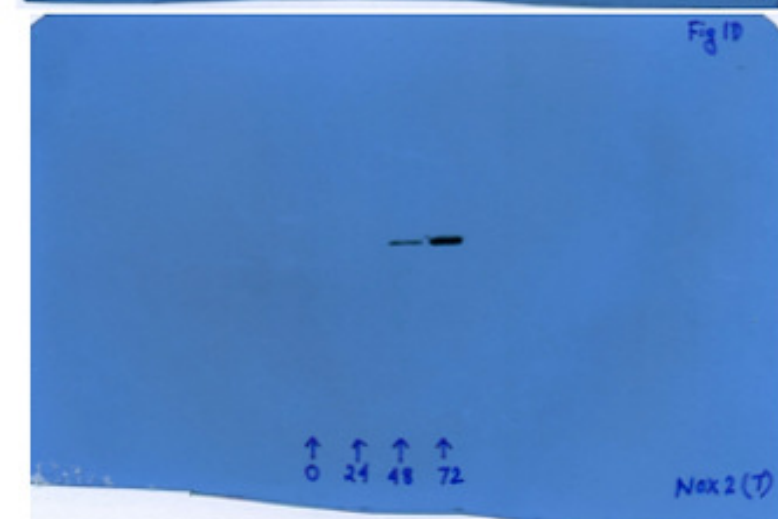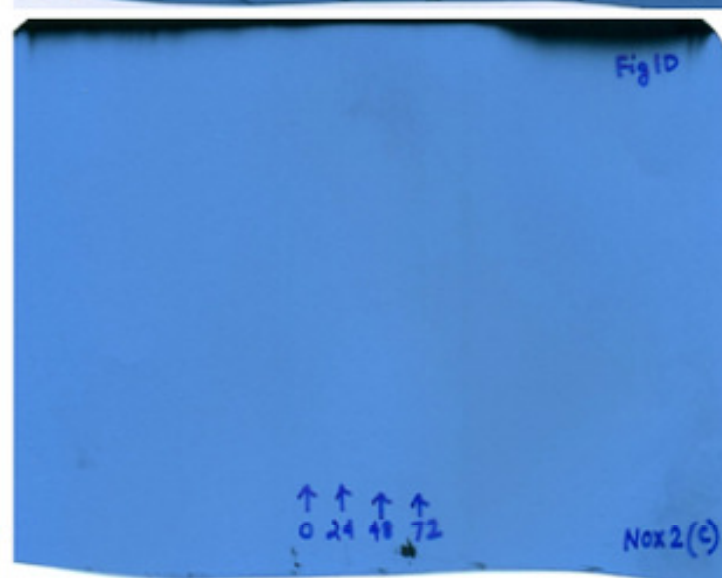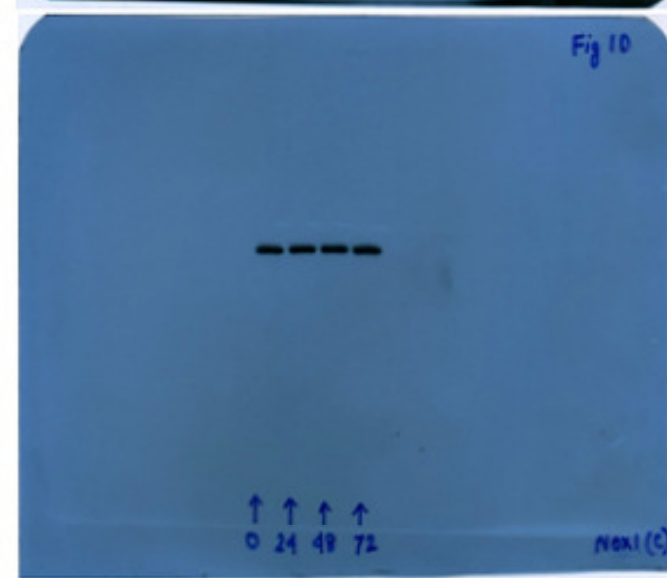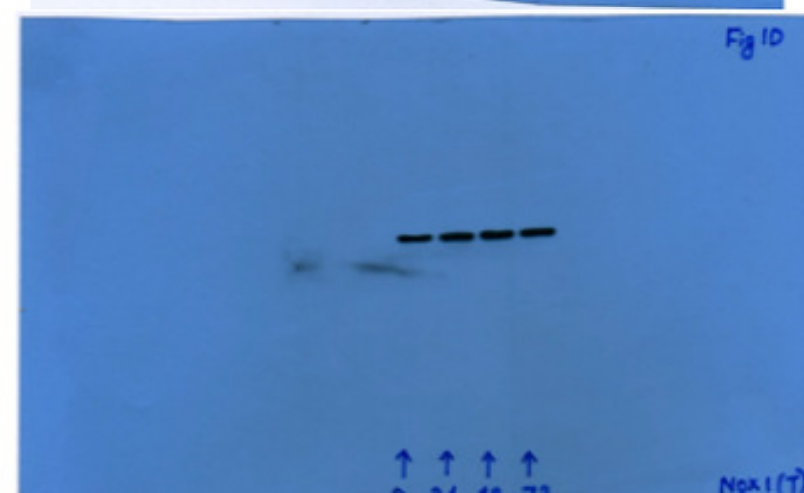

# Figure 3B

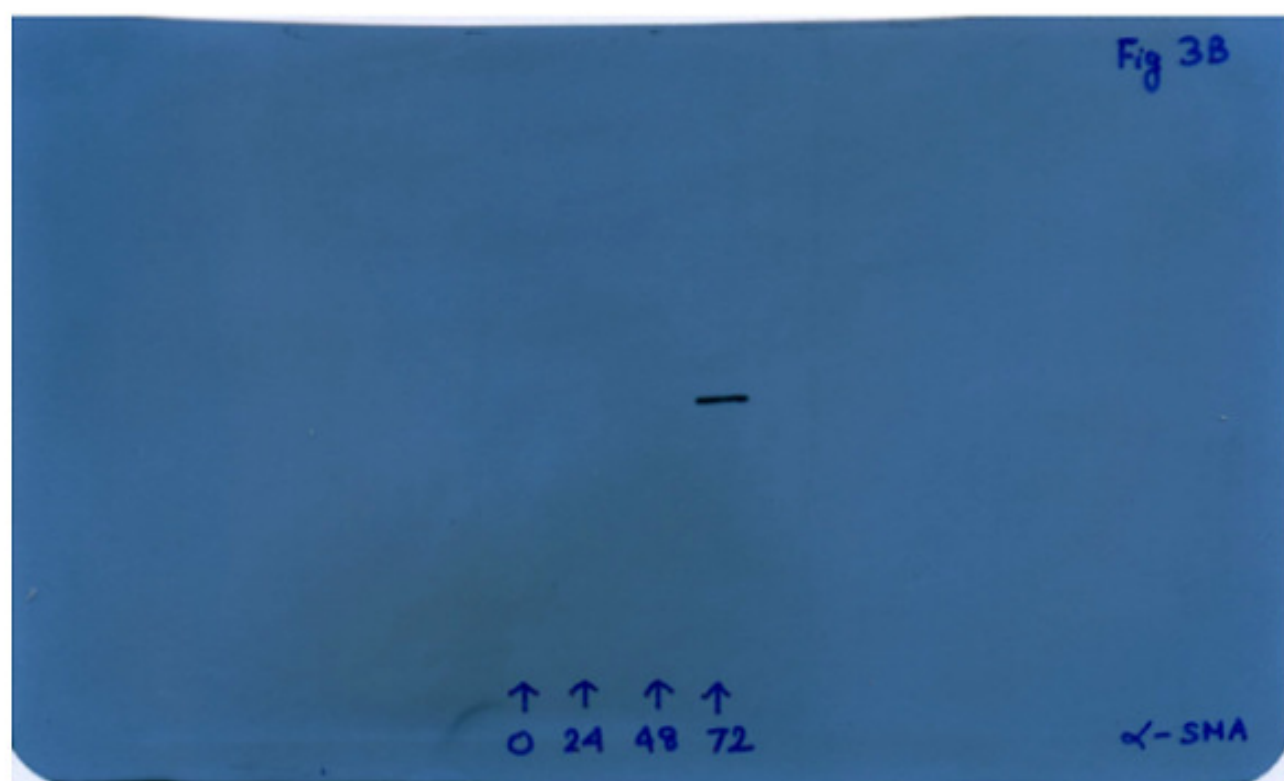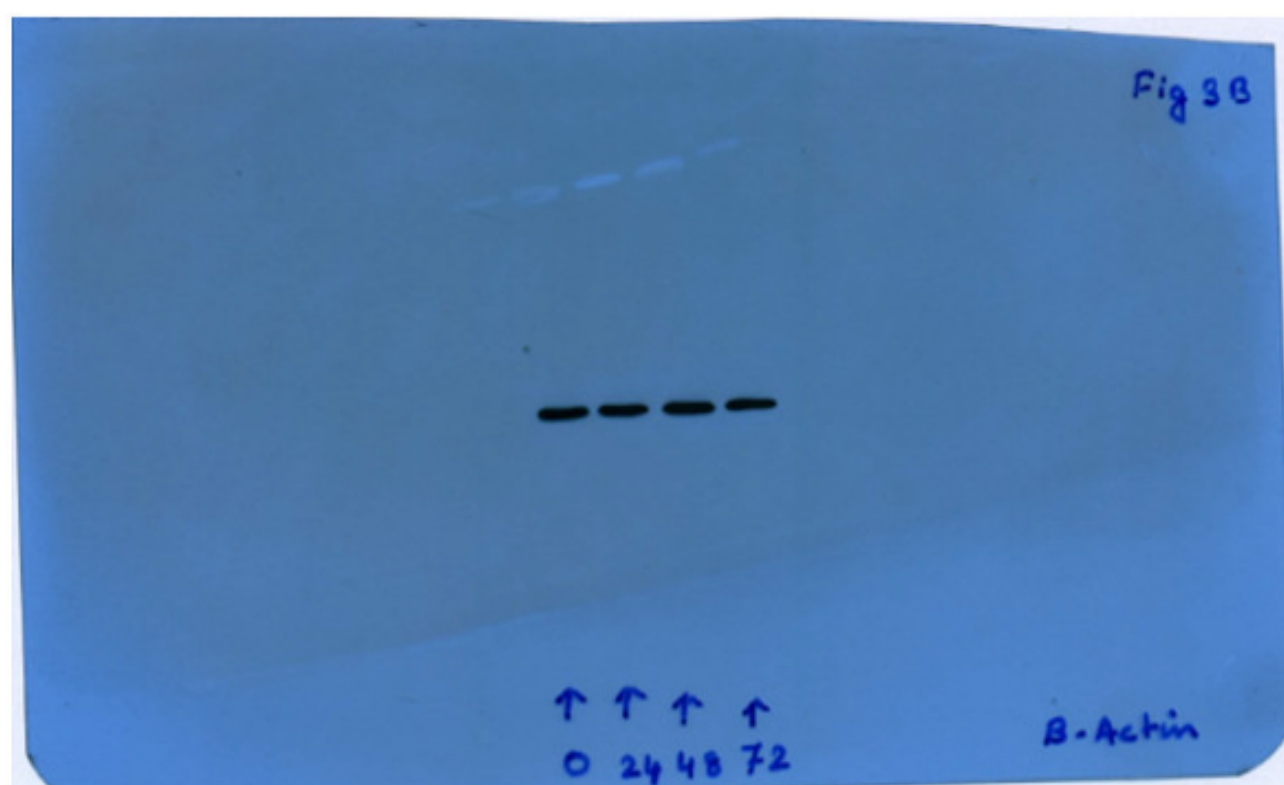

Supplement: S1 Raw Images — (PDF) [file pone.0236992.s001.pdf]
