# Supplementary material for: Expression of type I collagen in response to Isoniazid exposure is indirect and is facilitated by collateral induction of cytochrome P450 2E1: An in-vitro study
Source: PLoS One. 2020 Jul 31;15(7):e0236992. doi: 10.1371/journal.pone.0236992 (PMC7394448; doi:10.1371/journal.pone.0236992)
Supplement: Attachment — Submitted filename: Reviewers Comments plos one 2.doc [file pone.0236992.s002.doc]

Response to Reviewers

We would like to thank the academic editor and the reviewers for critical review of our manuscript and for their thoughtful comments and constructive suggestions, which help to improve the quality of this manuscript. Point wise answer of additional requirements and the comments of the reviewers are follows:

**Response in relation of additional requirements**:

1. “Please ensure that your manuscript meets PLOS ONE’s style requirements, including those for the naming”

**Answer**: We have carefully revised our manuscript as per format of PLOS ONE style.

2. “ORCID ID of the corresponding author”

**Answer**: The ORCID ID of the corresponding author, Dr. Abhijit Chowdhury, is orcid.org/0000-0001-9198-4436.

3. “PLOS ONE requires the authors provide the original uncropped and unadjusted images underlying blots or gel results reported in a submission’s figures or supporting information files”

**Answer**: Original uncropped and unadjusted images are submitted in a separate file “S1_raw_images” as per suggestion.

We are really sorry for not providing all the uncropped and unadjusted images of	blots for Figure 1D. Dr. Suman Santra left the department to join his Post	Doctoral research program in USA and Dr. Amal Santra on superannuation of his	service at IPGME&R, Kolkata, left the department and joined at JCM Center for	Liver research and Innovation Kolkata. His lab at IPGME&R was shifted to	JCM Center for Liver Research and innovation, Kolkata. During shifting his lab,	some of the documents were misplaced and missing. This is the reason; we are	unable to submit the uncropped and unadjusted images of blots of NOX1 (T) and	NOX2 for figure 1D. Hope, the academic editor as well as the reviewers will	sympathetically consider our case.

Further, in our lab, we carried out western blot development protocol using the	sensitive chemiluminescent substrate (Supersignal west Pico plus	chemiluminescent reagent (34580; Thermo Scientific, U.S.A) for the detection of	horseradish peroxidase (HRP) exposed to X-ray film. For gel electrophoresis and	western blot, we used Thermo Scientific SpectraTM Multicolor Broad Range	Protein Ladder (26634) which is a prestained protein ladder. This is a ready to	use ladder which can be directly loaded into gels. Since it is a prestained protein	ladder, we couldnt visualize any signal for the protein marker lane after	development protocol onto the exposed Xray film except for our desired protein	of interest.

4. “We noted that you have included the phrase “Data not shown” included in your manuscript. Unfortunately this does not meet the journal’s data sharing requirements. PLOS ONE does not permit references to inaccessible data.”

**Answer**: Our previous statement “Data not Shown” is corrected properly in the revised manuscript.

**Response of the comments of Reviewer 1**:

**Major comments**:

A. “The authors need to have every details of the reagents and chemicals used in each experiment in the manuscripts. …….. Moreover, the need to include the information on the respective primary antibody dilution in the Western blotting sub-section too”.

**Answer**: As per suggestion of the reviewer, details of the reagents and chemicals are provided in the revised manuscript along with catalogue numbers and vendors. Moreover, dilutions of the primary antibodies are mentioned in the revised text.

B. “In the biochemical assays sub-section of the materials and methods, The authors do not elaborate on the technique used to use different parameters like GSH, TBARS, protein content, CYP2E1, GPx, NOX”

**Answer**: As per suggestion of the reviewer, we elaborate all the biochemical techniques used in the revised text.

C. The authors have performed the Sircol Collagen assay and presented in the result. However, there is no mention of the assay technique in the materials and methods section”.

**Answer**: Sircol Collagen assay technique is mentioned in the materials and methods section in the revised text.

**Minor comments**:

i) “Some of the paragraphs or portions of paragraphs are underlined within the manuscript. Is it done deliberately, what does it mean? I will suggest that the authors be consistent in their manuscript”.

**Answe**r: Necessary changes are made in the revised manuscript as per suggestion of the reviewer.

ii) The authors indicate in the statistical analysis that all the experiments have were replicated at least five times. None of the individual figures possess any information on the number of replicates used for each experiment. I will suggest that the authors provide the exact number of experimental replicated used to prepare the quantification graphs in each figure”.

**Answer**: Number of times the experiments were conducted is inserted within the figure legends.

**Response of the comments of Reviewer 2**:

**Question 1:** “In figure 1D, the expression of NOX was detected by western blot. What are NOX1 (T) and NOX1 (C)? The results and figure legend didn’t explain these items.

The results need to show the expression of NOX in untreated cells. After INH treatment, there is not the obvious increase in NOX1 expression between 24 and 72 h. But due to lack of results in the untreated cells, I didn’t know the change in NOX1 expression after INH treatment compared to untreated control”.

**Answer**: In figure 1D, NOX1 (C) and NOX1 (T) are explained in the figure legends. NOX1 (C) is untreated control and NOX1 (T) is INH treated cells. After treatment of INH, increase of NOX1 expression between 24 and 72 hours exhibited no change. There is no difference in expression of NOX1 between control [NOX1 (C)] and INH treated cells [NOX1 (T)].

**Question 2:** In Figure 4A, the results showed the CYP2E1 activity was increased after INH, PY or INH+PY treatment. Could the authors also show the results using western blot about the expression of CYP2E1 after INH, PY or INH+PY treatment?

**Answe**r: As per suggestion of the reviewer, expression of CYP2E1 by western blot after INH, PY and INH+PY treatment is provided in the text and in figure 4D.

**Question 3:** CYP2E1 is expressed mainly in hepatocytes and generate ROS. Accumulation of ROS serves as a driving force for HSC. ……………the authors used pyrazole (PY) pre-treated HSC to increase the expression of CYP2E1. … affect the HSC activation in vitro culture assay.

**Answer**: Since it is a short term study, to enhance CYP2E1 activity in LX2 cells, we have used PY to induce CYP2E1.

**Question 4:** The results showed INH or PY treatment can induce HSC activation. Upon activation HSCs express a combination of MMPs and TIMPs to remodel the ECM and facilitate the progression of liver fibrosis. How about the proliferation of HSC and MMP expression after INH or INH+PY treatment?

**Answe**r: As per suggestion of the reviewer, proliferation of HSC cells after INH or PY+INH treatment is inserted within the revised text. Further, results of MMP 2 amd MMP 9 are also provided in Table 2 as well as in the text.

**Question 5:** In figure 2C, the results showed pretreatment of LX2 cells with anti-oxidant or NOX inhibitor can reduce the intracellular ROS formation in response to INH treatment. How about the effect of CYP2E1 inhibitor in the same experiment?

Since the authors demonstrated that INH induced CYP2E1 expression can activate HSC, whether the CYP2E1 inhibitor can reverse the effect in response to INH treatment?

**Answer**: ROS generation in presence of CYP2E1 inhibitor was significantly reduced which is provided in the text and figure 2C.

Activation of LX2 cells in presence of CYP2E1 inhibitor was markedly reduced which is provided in the text and figure 3C.
